# Disrupted metabolic signatures in amniotic fluid associated with increased risk of intestinal inflammation in cesarean section offspring

**DOI:** 10.3389/fimmu.2023.1067602

**Published:** 2023-01-24

**Authors:** Yongjie Liu, Jun Zhang, Liping Feng

**Affiliations:** ^1^ Ministry of Education and Shanghai Key Laboratory of Children’s Environmental Health, Xinhua Hospital, Shanghai Jiao Tong University School of Medicine, Shanghai, China; ^2^ Department of Obstetrics and Gynecology, Duke University School of Medicine, Durham, NC, United States

**Keywords:** cesarean section, vaginal delivery, amniotic fluid, multi-omics, intestinal inflammation

## Abstract

**Introduction:**

Children born by cesarean section (CS) are at a greater risk of inflammatory bowel disease (IBD). However, the mechanisms underlying the association are not yet well understood. Herein, we investigated the impact of CS delivery on colonic inflammation and mechanisms underlying these effects in offspring.

**Methods:**

CS mice model and dextran sulfate sodium (DSS)-induced colitis model were constructed and used to analyze the impact of CS on the development of colitis. Colonic tight junction markers and epithelium differentiation markers were analyzed by quantitative real-time polymerase chain reaction (qRT-PCR). Levels of zonulin in serum were detected by enzyme-linked immunosorbent assay (ELISA). Immune cells in colon were analyzed by flow cytometry. Metabolic profiling between human vaginal delivery (VD) and CS AF were analyzed by using mass spectrometry. Transcriptome changes between VD AF- and CS AF-treated human intestine epithelial cells were analyzed by RNA-sequencing. A multi-omics approach that integrated transcriptomics with metabolomics to identify the pathways underlying colonic inflammation associated with delivery modes. Then, the identified pathways were confirmed by immunoblotting and ELISA.

**Results:**

Mice pups delivered by CS exhibited a defective intestinal homeostasis manifested by decreased expression of tight junction markers of *ZO-1* and *Occludin* in the colons, increased levels of zonulin in serum and dysregulated expression of intestinal epithelium differentiation markers of *Lysozyme*, *Mucin2*, and *Dipeptidyl peptidase-4.* CS pups were more susceptible to DSS-induced colitis compared to VD pups. The proportion of macrophage, dendritic cells (DCs), and natural killer cells (NKs) in the colons were altered in an age-dependent manner compared with pups born naturally. The metabolites in AF differed between CS and VD cases, and the CS AF-induced differentially expressed genes (DEGs) were significantly enriched in pathways underlying IBD. Signal transducer and activator of transcription 3 (STAT3) signaling was downregulated in NCM460 intestinal epithelial cells by CS AF compared to VD AF and in colon of CS pups compared to VD pups. Deficiency in metabolites like vitamin D2 glucosiduronate in CS AF may attribute to the risk of inflammatory intestine through STAT3 signaling.

**Conclusion:**

Our study provides a novel insight into the underlying mechanisms of CS-associated intestinal inflammation and potential prevention strategies.

## Introduction

Cesarean section (CS) may be a lifesaving surgical intervention for an infant or mother who is at significant risk during childbirth. CS rates are increasing globally (1), and an overall hospital-based CS rate in China was 41.1% in 2016 (2, 3). Notably, the large increases in CS use often result from non-medical indications. Epidemiological studies have suggested that children born by CS are at greater risk of developing chronic inflammatory diseases, including inflammatory bowel disease (IBD), which comprises ulcerative colitis (UC) and Crohn’s disease (CD) (4). In a national cohort of *Danes*, researchers found that individuals born by CS were at an overall 14% increased risk of developing IBD before age 36 years compared with vaginally delivered peers, a risk increase that was most pronounced (29%) in children aged less than 15 years (4). A register-based cohort study in *Australia* reported that elective CS was significantly associated with increased risk of CD before age 16 (5). A meta-analysis of pooled data also indicates that CS is a risk factor for CD (6). However, there are major challenges in demonstrating causality considering the confounding factors, such as maternal health status, use of antibiotics, and formula feeding. Additionally, the mechanisms underlying the association are not yet well understood. It is imperative to investigate the mechanisms by which CS leads to an increased risk of intestinal inflammatory disease because it may facilitate the development of prophylactic strategies.

Evidence is emerging that intrauterine exposures have far-reaching effects on the lifelong health of infants, and the birth process and intrapartum interventions can also affect normal physiological or pathophysiological development (7). A greater risk of impaired immune responses has been significantly associated with CS birth in humans (8, 9). Previous animal studies have demonstrated that the fetal intestine is a potential site for the initiation of immune activation due to the existence of organized intestinal lymphoid structures (10–13). During fetal development, amniotic fluid (AF) is in direct contact with the gastrointestinal tract, whose components are in continuously change with increasing gestational age (14). A CS birth is absent in marked hormonal changes, regular contractions, and mechanical stimuli, compared to a vaginal delivery (VD) (7, 15), and these extraordinary events shift the composition of AF. However, the role of AF in the development of intestinal mucosa inflammation in CS infants is not clear.

The aim of this study was to investigate the impact of CS delivery on colonic inflammation and mechanisms underlying these effects in offspring. CS mice model and dextran sulfate sodium (DSS)-induced colitis model were constructed and used to investigate the causality of CS on colitis. Intestinal homeostasis was assessed by detecting the gut permeability, intestinal epithelium differentiation markers, and alteration of immune cells in colons of mice delivered by CS compared to VD. Metabolic profiling, the measurement of the complement of low-molecular-weight metabolites and their intermediates that reflects the dynamic response to physiological stimuli (such as labor) enables us to identify biomarkers and potential intervention strategies. We characterized the differential metabolic profiles of AF collected from women who had VD or CS using gas chromatography- and liquid chromatography-tandem mass spectrometry (GC- and LC-MS/MS), and then performed transcriptome analysis to compare gene expression between human intestine epithelial cells treated with these VD or CS AF samples. Finally, a multi-omics approach integrating transcriptomics and metabolomics to identify the underlying pathways involved in intestinal inflammation dictated by birth delivery modes.

## Materials and methods

### CS mice and DSS colitis model

Eight-week-old C57BL/6 mice were purchased from the Shanghai Slac Laboratory, maintained under a 12-hour light cycle and fed autoclaved chow to acclimate for 1 week. Adult female mice were cohabited with male mice at 2:1 ratio, and separated into foster, CS (n=6) and VD (n=6) dams. On gestational day E19, CS was performed, and litters were transferred to a foster mom within 30 min. VD litters were also fed by a foster mom at day 0. Pups of foster mom were removed from the cages before foster pups were added. The survivability of the fostered pups was recorded for each litter. One pup was collected randomly from each of the CS and VD litters at day 10, day 20, and day 30, respectively. Then colon tissues were collected for preparing the single-cell suspensions and total RNA extraction.

To establish the DSS-induced colitis model, eight-week-old CS and VD mice were treated with 2.5% (w/v) DSS (M.W. = 36,000-50,000 Da; MP Biomedicals) in their drinking water for seven days followed by regular access to water. Body weight, diarrhea, and other changes in feces of mice were recorded every day, and disease activity index (DAI) scores were calculated as described in **Supplementary Table S1**. All animal care and experimental protocols performed were approved by the Institutional Animal Care and Use Committee (IACUC) of Xinhua Hospital Affiliated to Shanghai Jiao Tong University School of Medicine.

### Human amniotic fluid and cord blood collection

Women with healthy pregnancies were included as study participants. AF samples were collected from seven non-indicative prelabor CS by maternal request and seven VD mothers with an uncomplicated singleton pregnancy. AF was centrifuged at 350×g, and the supernatant was immediately stored at -80°C until use. Briefly, an abdominal fenistil incision was made by an obstetrician through the abdominal and uterine muscles without cutting into the amnion membrane. A blunt end insertion with a catheter was made into the amnion membrane, and the CS AF was aseptically suctioned into a MediVac Container. VD AF was collected from expectant mothers with regular contractions and cervical dilation (≥ 3cm) using a sterile catheter after rupture of membranes. There were no visible blood or meconium contaminations in the AF samples. Umbilical cord blood samples of VD and CS subjects were collected, centrifuged for serum, and stored at -80 °C for future use. The general characteristics of the study participants are presented in **Supplementary Table S2**. Pregnant women with symptoms or clinical evidence of chronic hypertension, diabetes, chronic renal disease, human immunodeficiency virus infection or other sexually transmitted infections were excluded. The studies were approved by the Ethics Committees of Xinhua Hospital Affiliated to Shanghai Jiao Tong University School of Medicine (XHEC-C-2015-036). A written informed consent was received from participants prior to the inclusion in study.

### Metabolic profiling analyses of AF samples

AF samples collected from seven CS and seven VD mothers were subjected to GC- and LC-MS/MS analyses as described previously (16, 17). The acquired raw data were analyzed by the Progenesis QI software (Waters Corporation, Milford, USA) using the following parameters: precursor tolerance at 5ppm, fragment tolerance at 10ppm, and product ion threshold at 5%. Metabolites were identified based on public databases such as the Human Metabolome Database (HMDB), Lipidmaps (v2.3), and METLIN. Principle component analysis (PCA) and (orthogonal) partial least-squares-discriminant analysis (OPLS-DA) models were carried out to visualize the metabolic alterations among experimental groups. A permutation test (n=200) was performed to validate the model and avoid over fitting. Hierarchical cluster analysis (HCA) was applied on Pearson distances using PermutMatrix (18). Differentially expressed metabolites (DEMs) contributing to the separation were identified using variable importance in the projection (VIP) values obtained from the OPLS-DA model and *p* values from a two-tailed Student’s t-test on the normalized peak areas from different groups, where metabolites with VIP > 1.0, Fold Change >2, and *p* < 0.05 were considered as differential metabolites.

### Cell culture and treatment

Human colon mucosal epithelial cell line NCM460 was a kind gift from professor Fuping You at the Department of Immunology, Peking University Health Science Center. Cells were cultured in RPMI 1640 medium supplemented with 10% (v/v) fetal bovine serum and 100 units penicillin/streptomycin (Gibco, USA) at 37°C with 5% CO_2_ atmosphere in a humidified incubator. Cells were negative for mycoplasma. After sterile filtration with using a 0.22 μm filter (Millipore), AF samples were added to the cell culture medium to the final 10% (v/v) concentration. Three AF samples were selected randomly from each group (VD and CS) and used for NCM460 cell treatment. After treatment with AF for 48h, cells were collected and used for further experiments. All experiments were carried out in duplicate.

### RNA extraction and library construction

After treatment with AF samples for 48 h, NCM460 cells were collected for transcriptomic analysis. After washing with sterile PBS, 1mL TRIzol regent (Invitrogen, USA) was added to the cells for total RNA extraction (Ambion, USA). RNA integrity was evaluated using the Agilent 4200 TapeStation (Agilent Technologies, USA). All samples in this study had an RNA Integrity Number (RIN) ≥ 9.6. For library construction, ribosomal RNA was removed and poly (A) + mRNA was purified with magnetic Oligo-dT beads (Invitrogen, USA). The purified mRNA was fragmented (200bp-300bp) using divalent cations under elevated temperature. These short fragments were copied into the first-strand cDNA using random hexamer primers and M-MuLV reverse transcriptase (Invitrogen, USA). Second-strand cDNA was synthesized by adding DNA Polymerase I and RNase H. The products were then enriched by PCR amplification for 15 cycles and separated by AMPure XP beads (BECKMAN COULTER, USA). The amplicons sized of 300~400 bps were collected and purified. The six constructed cDNA libraries (three VD-treated and three CS-treated) were sequenced using the Illumina HiSeqTM 2500 platform (OEbiotech Co., Ltd., Shanghai, China).

### RNA-seq data analysis

Trimmomatic (19) was used for cleaning the raw reads, including the removal of adapter sequences (cutadapt v1.2.1) and low-quality sequences (reads with ≥ 5% undetermined base pairs, denoted as ‘N’). The high-quality reads were mapped to GRCh37/hg19 using HISAT2 (Hierarchical Indexing for Spliced Alignment of Transcripts) (20). Count files of the sequencing reads were generated by the HTSeq-count script (21). After combining all read counts from each sequenced sample into a count file, differentially expressed genes (DEGs) analysis was carried out using DESeq2 packages (version 1.23.3) following standard normalization procedures (22). Genes with less than five total counts were removed and not used in subsequent analyses. In this study, adjusted *P* value < 0.05 and Fold Change >2 were set as the threshold for significant differential expression. HCA of DEGs was performed to explore transcripts expression patterns. Gene Ontology (GO) enrichment and Kyoto Encyclopedia of Genes and Genomes (KEGG) pathway enrichment analysis of DEGs were performed using R packages based on the hypergeometric distribution.

### Quantitative real-time polymerase chain reaction

Total RNA was isolated from cells using TRIzol reagent (Invitrogen, USA), and 1μg of total RNA was used for cDNA synthesis with PrimeScript™ RT reagent Kit with gDNA Eraser (Takara, Japan). Next, qRT-PCR was performed to confirm DEGs with *GAPDH* as the reference gene. Amplification of specific transcripts was confirmed by melting curve profiles at the end of each PCR cycle. The relative value to controls was determined by the 2^-ΔΔCT^ method (23). The primer sequences are listed in **Supplementary Table S3**.

### Flow cytometry

Fresh colon tissues of CS and VD mice was isolated and rinsed in cold PBS. The tissue was opened longitudinally and sliced into small fragments (~0.5cm in length). The tissue was incubated in PBS (with 5mM EDTA, 2mM DTT and 0.2% BSA) and then shaken vigorously for 20 min at 37°C. The tissue was then washed with fresh EDTA-PBS for three times. After washing, tissues were incubated in RPMI1640 supplemented with 0.5mg/mL each of neutral protease, collagenase and DNase l with vigorously sharking at 37°C for 1h. The single cell suspension was then passed through a 40μm filter and centrifuged at 300×g for 10min. Cell plates were resuspended in cold PBS and stained with fluorochrome-conjugated anti-mouse antibodies (Biolegend, San Diego, CA, USA) against macrophage (CD11b, F4/80), dendritic cells (DCs) (CD11c, MHC II), and NK cells (CD3, NK1.1) **(** **Supplementary Table S4** **)**.

### Immunofluorescence staining

Colon tissues from VD and CS pups were fixed with 4% paraformaldehyde and embedded in paraffin, and cut into 3-μm sections. Vector Antigen Unmasking Solutions (VECTOR LABORATORIES) were used for antigen retrieval. Slides were permeabilized with 0.5% Triton X-100 for 1 h at room temperature and then incubated at 4°C for 16 h with primary antibodies (**Supplementary Table S4**) at 2mg/mL followed by incubation with a secondary antibody at 37°C for 2 h. Sections were counterstained with 4,6-diamidino-2-phenylindole (DAPI) for nuclear staining. Images were captured using a Pannoramic MIDI digital scanner (3D HISTECH).

### Gut permeability detection in CS mice

Blood samples of CS and VD pups were collected and centrifuged for serum collection. The zonulin concentration in serum of CS and VD pups was determined by an enzyme-linked immunosorbent assay (ELISA) kit (DY4409-05, R&D Systems) using the double antibody sandwich ABC-ELISA method. All tests were carried out in duplicates in a blinded manner.

### Immunoblotting analyses

Immunoblotting was carried out by standard procedures. Briefly, protein samples were extracted by using RIPA lysis with protease inhibitor cocktail and concentrations were determined using a BCA Protein Assay kit (EpiZyme, China). Then protein samples eluted with SDS-PAGE sample loading buffer were boiled at 100°C for 5min and then loaded to gels. After electrophoresis, proteins were transferred to a 0.22-μm PVDF membrane (Millipore). The membrane was blocked with 5% skim milk powder (Merck) in TBST. After incubation with the following primary antibodies: anti-Occludin (sc-133256, Santa Cruz), anti-ZO1 (sc-33725), anti-Stat3 (sc-8019), anti-p-Stat3 (sc-8059), and anti-β-Actin (sc-47778) at a recommend dilution overnight at 4°C, and with m-IgGκ BP-HRP antibodies (sc-516102) for 2h at room temperature, the membranes were detected by enhanced chemiluminescence.

### Cytokines quantification

AF samples were centrifuged at 350×g and passed through a 0.22-μm filter (Millipore). Serum samples were collected by centrifugation from cord blood of CS and VD subjects. The levels of IL-6 in AF, and IL-10 and tumor necrosis factor-α (TNF-α) in serum of CS (n=7) and VD (n=7) subjects were measured according to a commercial ELISA kit (R&D Systems, USA).

### Statistical analysis

Statistical analyses and graphs were carried out using Prism 8 software (GraphPad Software). For all bar graphs, data were expressed as means ± standard error of mean (SEM). A two-tail student’s t-test was performed to analyze the differential in metabolites abundance, mRNA expression, and cell proportion between the VD and CS groups. *P* < 0.05 was considered statistically significant, and the *p*-value was adjusted by the Benjamini-Hochberg (BH) FDR correction.

## Results

### Pups delivered by CS had lower body weight, shortened colons, and dysregulated expression of intestinal epithelium differentiation genes

**Figure 1:**
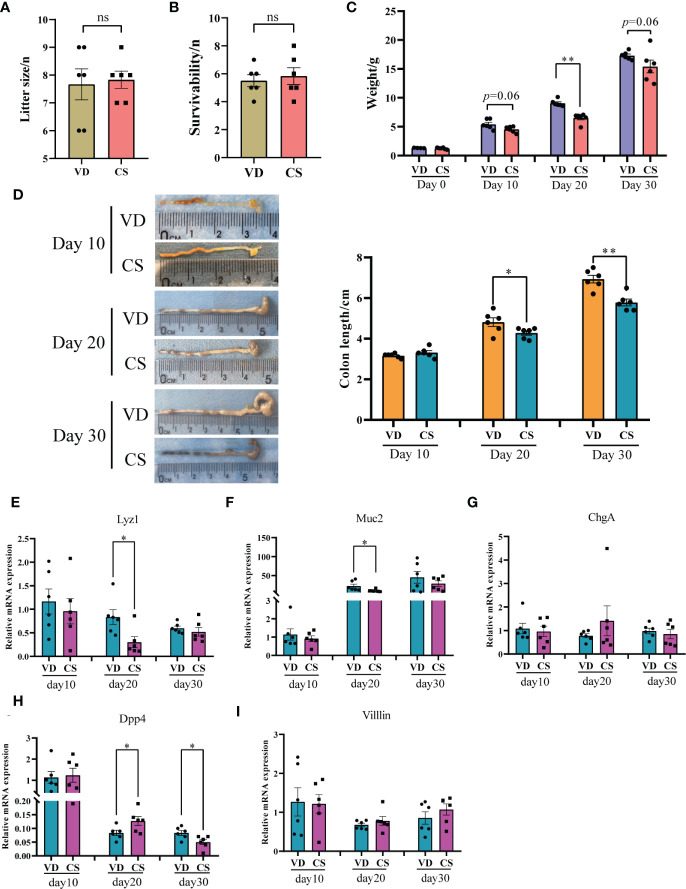
Body weight, the length of colons, and expression of intestinal epithelium differentiation genes in a murine model. Litter size **(A)** and the survivability of the fostered pups **(B)** between the cesarean section (CS) group (n = 6) and the vaginal delivery (VD) group (n = 6). **(C)**, body weight between pups delivered by CS (n = 6) and VD (n = 6) at day 0, day 10, day 20, and day 30 after birth. **(D)**, Colon length for CS-delivered (n = 6) compared with VD (n = 6) pups at day 10, day 20, and day 30 after birth. Relative mRNA expression of intestinal epithelium differentiation biomarkers, *Lysozyme* (*Lyz1*, **E**), *Mucin2* (*Muc2*, **F**), *Chromogranin-A* (*ChgA*, **G**), *Dipeptidyl peptidase-4* (*Dpp4*, **H**), and *Villin* **(I)** in colons of CS delivered pups (n = 6) compared with VD pups (n = 6). Significance was calculated with a two-tail student’s t test. Error bars represent SEM. *p < 0.05, **p < 0.01, ns indicates no significance.

After CS surgery, both CS pups and VD pups were transferred to a foster mom. There was no significant difference in litter size (**Figure 1A**) and the survivability of the fostered pups (**Figure 1B**) between the CS group and the VD group. The characteristics of CS and VD pups including body weight and sex were listed in **Supplementary Table S5**. No differences in birth weight (*p*=0.26) were found between pups delivered by CS vs. VD. The body weight of CS pups at day 20 was significantly lower than that of VD pups (*p*=0.002, **Figure 1C**). After adjusting for sex, there was still a significant difference of body weight between CS pups and VD pups at day 20 (β=2.46 (1.68-3.24), *p*=0.0002, **Supplementary Table S6**). Although a similar trend was also observed, no significances were found at day 10 (*p*=0.06) and day 30 (*p*=0.06) after birth (**Figure 1C**). CS pups had shorter colons compared with VD-delivered pups at day 20 and day 30 after birth, and no significances were found at day 10 (**Figure 1D**). Moreover, RT-qPCR results demonstrated that the gene expression of intestinal secretory cell markers, *Lysozyme* (*Lyz1*, **Figure 1E**) and *Mucin2* (*Muc2*, **Figure 1F**) were significantly decreased in colon of CS-delivered pups compared with VD pups at day 20. While intestinal absorptive cell maker, *Dipeptidyl peptidase-4* (*Dpp4*, **Figure 1H**) was markedly increased in colons of CS pups at day 20, but was decreased at day 30 compared to VD pups. No differences were found for *Chromogranin-A* (*ChgA*, **Figure 1G**) and *Villin* (**Figure 1I**).

### Pups delivered by CS exhibited a defective epithelial barrier and an increased susceptibility to DSS-induced colitis

**Figure 2:**
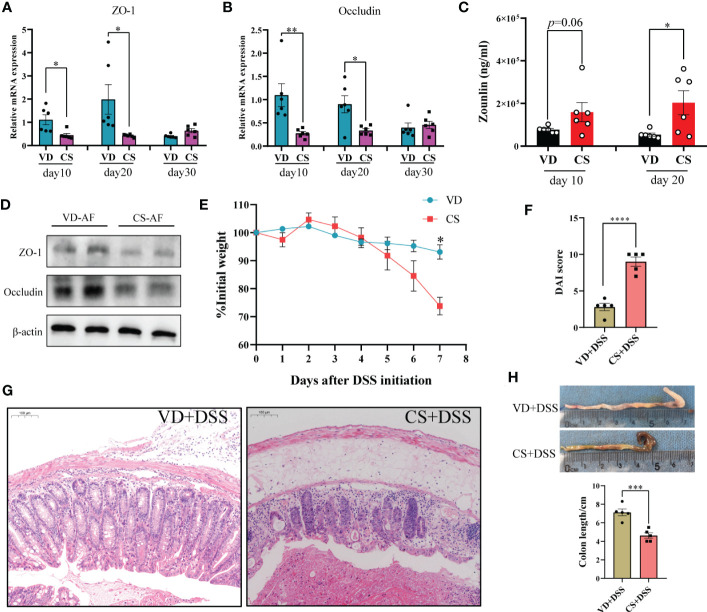
Pups delivered by CS exhibited a defective epithelial barrier and an increased susceptibility to DSS-induced colitis. Relative mRNA expression of tight junction protein 1 (ZO-1, **(A)** and Occludin **(B)** in colons of CS delivered pups compared with VD pups. **(C)**, Concentrations of zonulin were quantified by ELISA in serum of VD (n = 6) and CS (n = 6) pups at day 10 and day 20. **(D)**, Protein expression of ZO-1 and Occludin in CS amniotic fluid (AF)-treated NCM460 cells. **(E)** Weight loss compared to day 0 after administration of DSS in VD (n = 5) or CS (n = 5) pups for 7 days. **(F)** Disease activity index (DAI) scores for mice in VD and CS groups. **(G)** Representative H&E-stained colon sections of VD and CS mice after administration of DSS. **(H)** Representative images of colons of VD and CS mice after administration of DSS. Error bars represent SEM. *p < 0.05, **p < 0.01, ***p < 0.001, ****p < 0.0001.

RT-qPCR results showed that the gene expression of gut integrity biomarkers, tight junction protein 1 (*ZO-1*) and *Occludin*, was significantly downregulated in the colon of CS-delivered pups compared with VD pups at day 10 and day 20 (**Figures 2A, B** **)**. The concentration of the gut permeability biomarker zonulin, was notably increased in the serum of CS pups in comparison with VD pups at day 20 (**Figure 2C**), and also showed an increase trend at day 10 (*p*=0.06). Moreover, ZO-1 and Occludin were also down-regulated in CS AF-treated NCM460 cells (**Figure 2D**). An aggravated features of DSS-induced colitis were present in CS pups, including increased weight loss (**Figure 2E**), disease activity index (DAI) scores (calculated by body weight decreases + stool consistency + rectal bleeding) (**Figure 2F**), mucosal damage, and crypt destruction (**Figure 2G**), as well as a decreased colon length compared to VD pups (**Figure 2H**).

### The proportions of immune cells were altered in colon of pups delivered by CS

**Figure 3:**
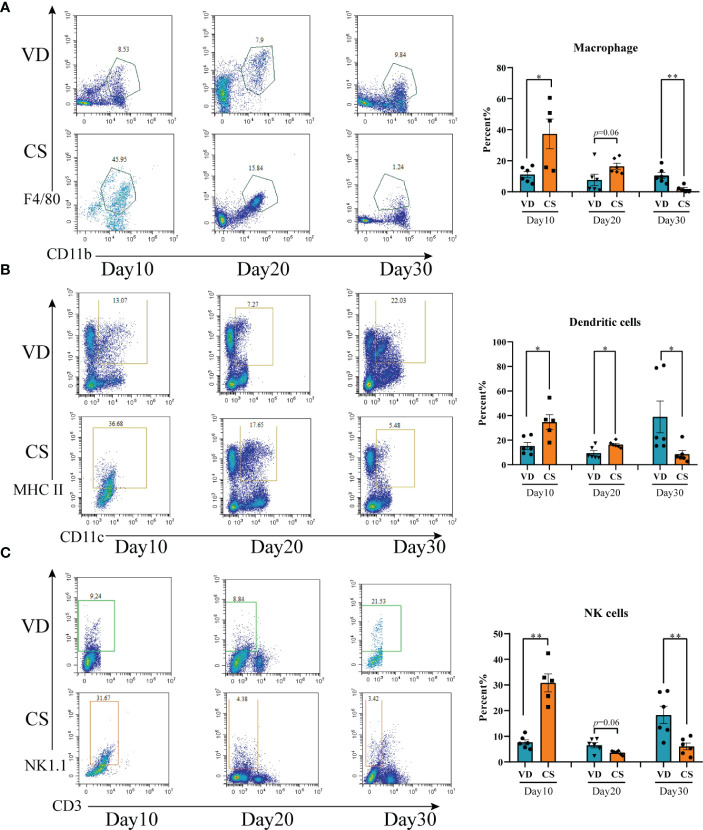
Cesarean section altered the proportions of intestinal immune cells in mice offspring. **(A)**, Representative flow cytometric analysis of macrophage from lymphocyte of colons from CS and VD pups at day 10, day 20, and day 30 after birth (n = 6). **(B)**, Representative flow cytometric analysis of dendritic cells from lymphocyte of colons from CS and VD pups at day 10, day 20, and day 30 after birth (n = 6). **(C)**, Representative flow cytometric analysis of NK cells from lymphocyte of colons from CS and VD pups at day 10, day 20, and day 30 after birth (n = 6). Significance calculated with a two-tail student’s t test. Error bars represent SEM. *p < 0.05, **p < 0.01.

A higher proportion of macrophage (**Figure 3A**), DCs (**Figure 3B**), and NK cells (**Figure 3C**) were present in colon in CS pups compared with VD pups at day 10 (*p*=0.02, *p*=0.01, *p*=0.004, respectively). Higher levels of DC cells (*p*=0.01), but not macrophage (*p*=0.06) and NK cells (*p*=0.06), were seen in CS pups vs VD pups at day 20. At day 30, lower levels of macrophage (*p*=0.005), DCs (*p*=0.03), and NK cells (*p*=0.009) were observed in CS pups compared with VD pups. Moreover, IF staining further demonstrated the changes of quantities of macrophage, DCs, and NK cells in colons of CS mice compared with VD mice at day 30 **(** **Supplementary Figure S1** **)**. These results suggest that CS is associated with an increased susceptibility to immune cells-mediated colonic inflammation.

### AF metabolites profile differed significantly in CS and VD

**Figure 4:**
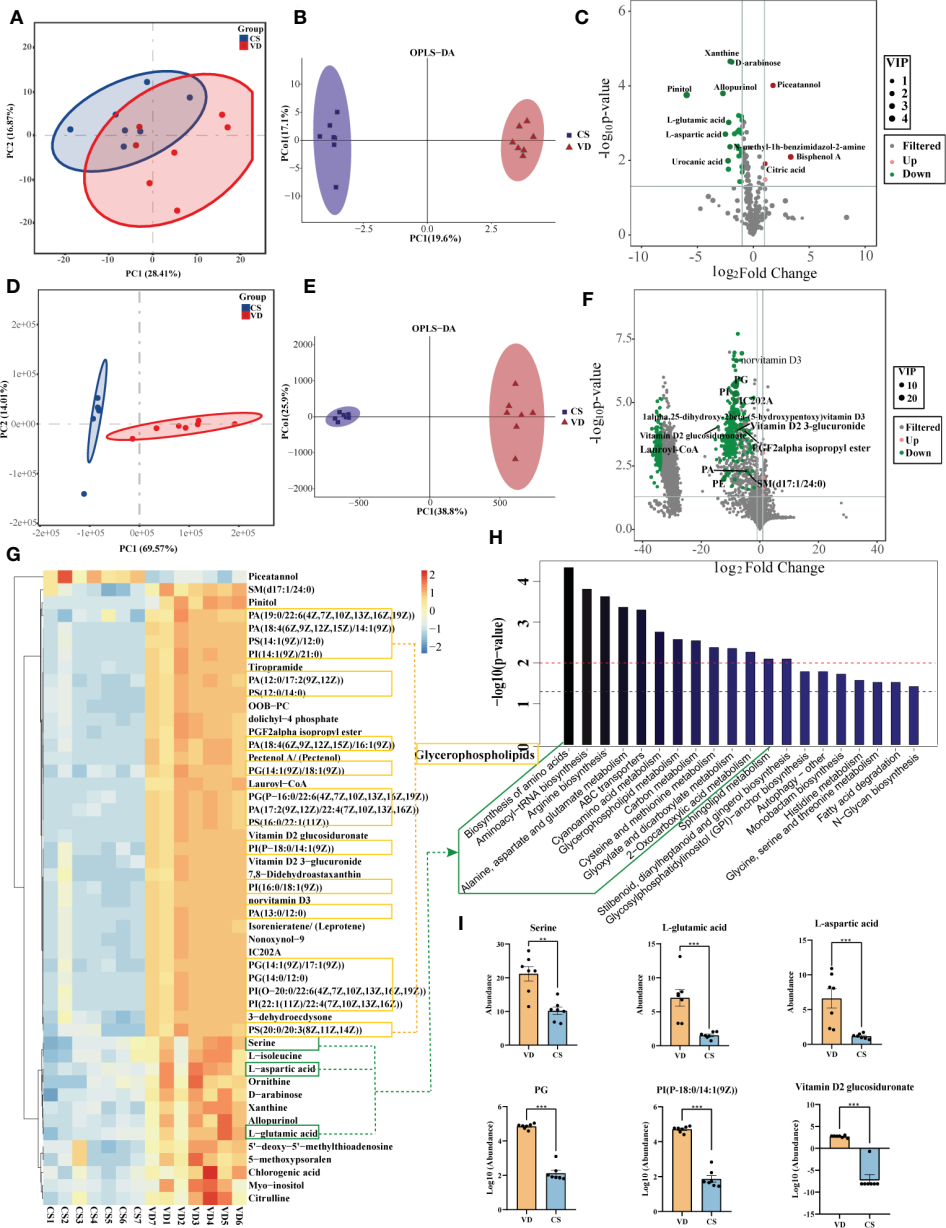
Metabolomics analysis for AF metabolites profiles in CS women compared with VD women. PCA **(A)** and OPLS-DA **(B)** score plots for differentiating the metabolites in each group by GC-MS/MS. **(C)**, Volcano plot showing the differentially accumulated metabolites in the CS AF (n = 7) and VD AF (n = 7) by GC-MS/MS. PCA **(D)** and OPLS-DA **(E)** score plots for differentiating the metabolites in each group by LC-MS/MS. **(F)**, Volcano plot showing the differentially accumulated metabolites in the CS AF and VD AF by LC-MS/MS. **(G)**, Hierarchical clustering analysis (HCA) of the metabolites that differed between the CS (n = 7) and VD (n = 7) subjects. **(H)**, Enriched KEGG pathways in CS AF group compared with VD AF group. **(I)**, Abundance comparisons of metabolites between CS AF group and VD AF group. Error bars represent SEM. **p < 0.01, ***p < 0.001.

Both GC-MS/MS and LC-MS/MS were performed to analyze the AF metabolic profiles between VD and CS subjects. The quality control (QC) samples in the PCA score plot overlapped, which indicates that samples behaved stably for the duration of the run. Using PCA and OPLS-DA, we found that the CS group was clearly distinct from VD group, demonstrating that metabolic disturbances exist between the two groups (**Figures 4A, B, D, E**). Based on the differential screening strategy, 62 differential metabolites were found in the CS AF compared with the VD AF by GC-MS/MS (**Figure 4C**), and 200 differential metabolites were identified by using the LC-MS/MS method (**Figure 4F**). The HCA of the metabolites that differed between the CS and VD subjects revealed three large clusters: (i) glycerophosphplipids, (ii) sterol lipids and prenol lipids, and (iii) fatty acyls, all of which were in lower abundance in CS AF than in VD AF (**Figure 4G**). The KEGG pathway enrichment analyses showed that these differentially presented metabolites were related to 1) biosynthesis of amino acids (L-aspartic acid, L-glutamic acid, serine, citric acid, citrulline, and L-isoleucine); 2) glycerophospholipid metabolism (PC, PE, and 2,3-Bis-O-(geranylgeranyl)glycerol 1-phosphate); 3) carbon metabolism (L-aspartic acid, L-glutamic acid, serine and citric acid); and 4) fatty acid degradation (Lauroyl-CoA, and trans,cis-Lauro-2,6-dienoyl-CoA) et al. (**Figure 4H**). Specifically, the metabolites with higher VIP values, such as PG, PI, PS, and Vitamin D2 glucosiduronate were at significantly higher levels in VD AF compared to CS AF sample (**Figure 4I**). Taken together, our data demonstrated that CS has a unique AF metabolome, suggesting that certain metabolites may be associated with fetal or infant intestinal development, as fetuses swallow AF constantly.

### Differentially expressed genes were identified in human intestinal mucosal epithelial cells treated by CS AF

**Figure 5:**
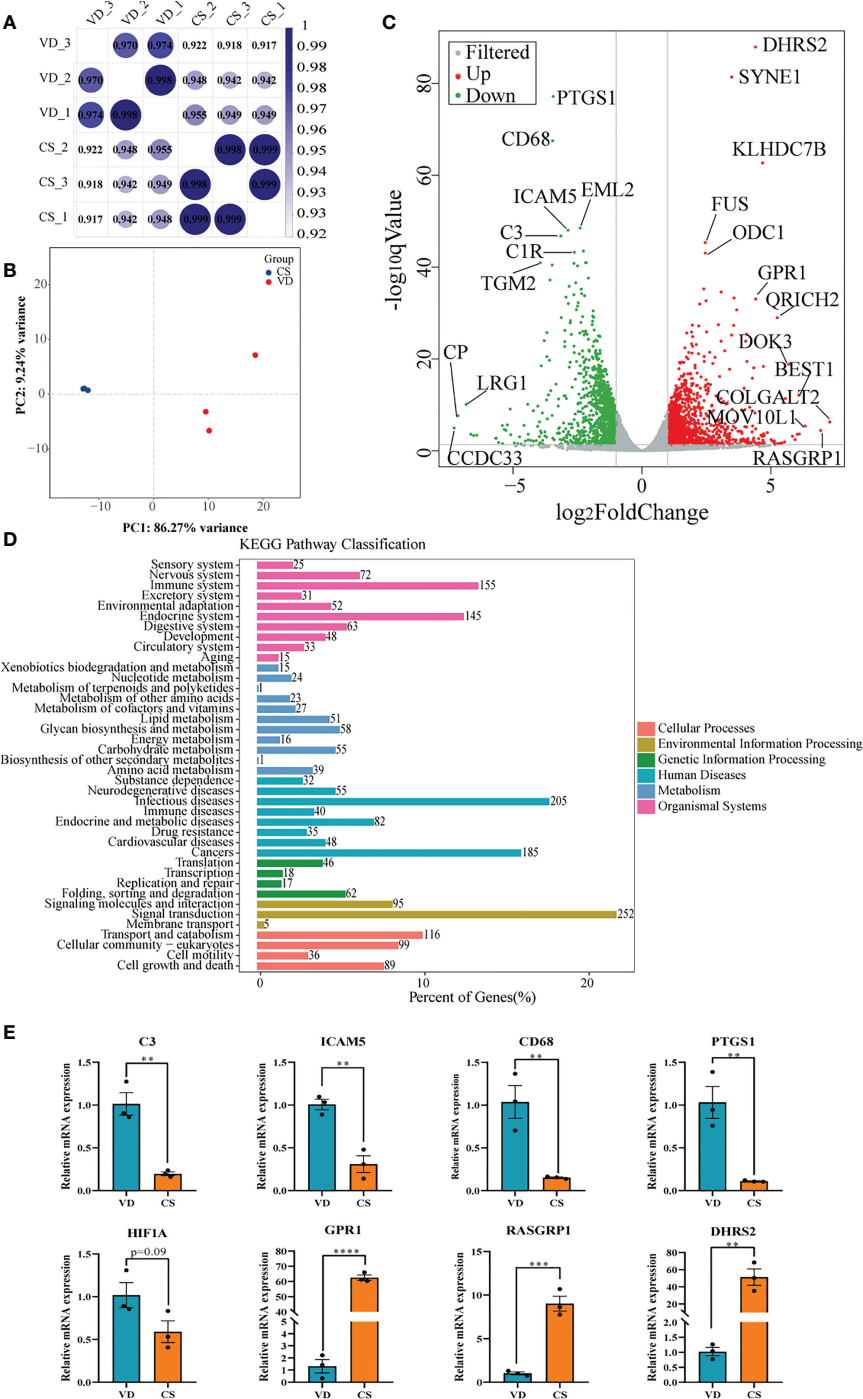
Transcriptome analyses of human intestinal epithelial NCM460 cells after CS AF stimuli. **(A)**, Correlation heatmap analysis between CS AF-treated cells (n = 3) and VD AF-treated cells (n = 3). **(B)**, PCA score plots for differentiating the genes in each group. **(C)**, Volcano plot showing the differentially expressed genes (DEGs) in the CS AF-treated group (n = 3) and VD AF-treated cells (n = 3). Red dots represent the DEGs up-regulated in the CS AF-treated cells, and green dots represent the DEGs down-regulated in the CS AF-treated cells. **(D)**, KEGG classification analysis of DEGs. **(E)**, Validation of DEGs between CS AF-treated group (n = 3) and VD AF-treated group (n = 3) using RT-qPCR. The change in fold-expression was calculated by the 2-∆∆CT method using GAPDH as a reference gene. Error bars represent SEM. **p < 0.01, ***p < 0.001, ****p < 0.0001.

Transcriptomic analysis was performed to investigate the effect of AF samples on intestinal mucosal epithelial cells. A heatmap was used to analyze the correlation between the treatment groups and biological replicates, with the correlation increasing as the value approaches 1.00. As shown in **Figures 5A, B**, there were significant differences between the VD-treated and CS-treated cells, and the grouping is clear. The volcano maps were drawn to intuitively represent the differential gene distribution between the VD- and CS-treatment groups. According to the cut-off values (Fold Change ≥ 2, FDR < 0.05), there were 2,516 DEGs between VD and CS treated cells, of which 1,275 were upregulated and 1,241 were downregulated (**Figure 5C**). KEGG classification analysis indicated that 252 (26.06%) DEGs were associated with signal transduction, 205 (21.2%) DEGs were associated with infectious diseases, and 155 (16.03%) DEGs were associated with the immune system (**Figure 5D**). The genes with a great fold change including complement C3 (*C3*), intercellular adhesion molecule 5 (*ICAM5*), CD68 molecule (*CD68*), prostaglandin-endoperoxide synthase 1 (*PTGS1*), hypoxia inducible factor 1 subunit alpha (*HIF-1A*), G-coupled protein receptor 1 (*GPR1*), RAS guanyl releasing protein 1 (*RASGRP1*), and dehydrogenase/reductase 2 (*DHRS2*) were confirmed by RT-qPCR (**Figure 5E**).

### Pathways underlying IBD were significantly enriched in CS AF-treated human intestinal mucosal epithelial cells

**Figure 6:**
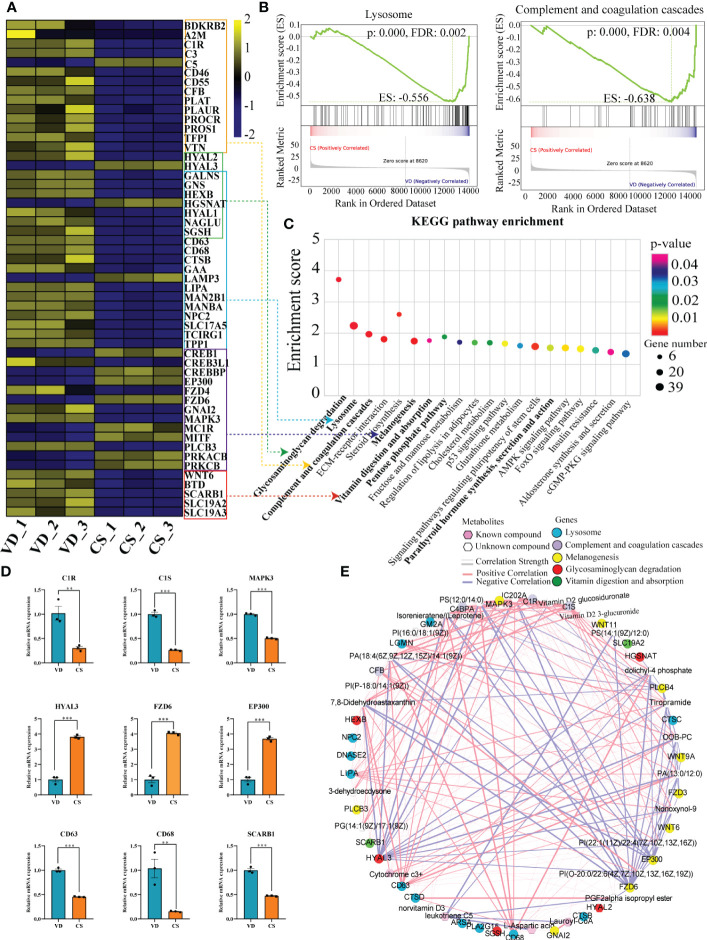
Correlation analyses of differential transcripts and metabolites. **(A)**, Heat map comparing the expression of DEGs between CS AF- (n = 3) and VD AF-treated (n = 3) human intestinal epithelial NCM460 cells. In the heat map, yellow represents high expression, and blue represents low expression. **(B)**, Gene Set Enrichment Analysis (GSEA) for the potential altered pathways between CS AF- and VD AF-treated NCM460 cells. **(C)**, KEGG pathway enrichment analysis indicated that the lysosome, glycosaminoglycan degradation, and complement and coagulation cascades were significantly enriched by DEGs. **(D)**, Validation of DEGs involved in above pathways between CS AF- (n = 3) and VD AF-treated (n = 3) group by using RT-qPCR. The change in fold-expression was calculated by the 2-∆∆CT method using GAPDH as a reference gene. Error bars represent SEM. **p < 0.01; ***p < 0.001. **(E)**, Correlation-based joint analysis between the representative differentially expressed metabolites (DEMs) and DEGs.

The imminent development of Crohn’s disease is associated with changes in the complement cascade, lysosomes, innate immune response, and glycosaminoglycan metabolism. In this study, KEGG pathway enrichment analysis demonstrated that the lysosome, glycosaminoglycan degradation, and complement and coagulation cascades were significantly enriched by DEGs (**Figure 6A**). Lysosome, as well as complement and coagulation cascades pathways were also identified by gene set enrichment analysis (GSEA) (**Figure 6B**). Moreover, other IBD-related pathways like parathyroid hormone synthesis, secretion and action, melanogenesis, vitamin digestion and absorption, and pentose phosphate pathway were significantly enriched in CS AF-treated intestinal mucosal epithelial cells (**Figure 6C**). Validation on the relative expression levels of key genes related to these pathways was performed by qRT-PCR (**Figure 6D**). Furthermore, a correlation-based joint analysis between the representative DEMs and DEGs was conducted. Positive correlations were observed between IBD relevant metabolites (Lauroyl-CoA, PG, PI, PS, and Vitamin D2 glucosiduronate et al.) and DEGs (mitogen-activated protein kinase 3 (*MAPK3*), hyaluronidase 3 (*HYAL3*), frizzled class receptor 6 (*FZD6*), E1A binding protein p300 (*EP300*), complement C1s (*C1S*), *CD63*, and *CD68* et al) (**Figure 6E**). These results indicate that the differential metabolic profiles in CS AF might contribute to the development of IBD in offspring.

### CS abrogated IL-6 signaling through inhibition of both STAT3 protein and STAT3 phosphorylation

**Figure 7:**
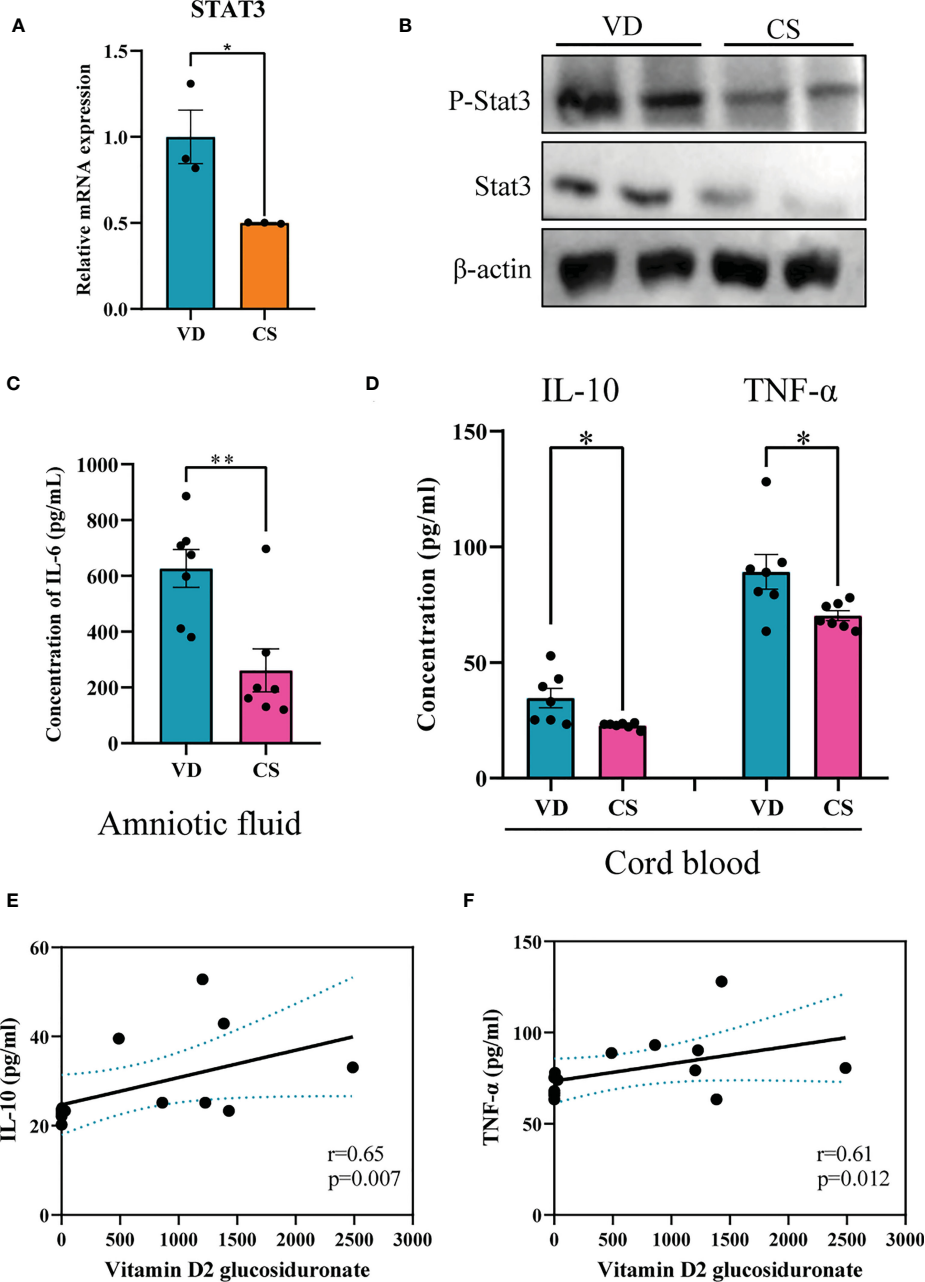
CS abrogated IL-6 signaling through inhibition of both STAT3 protein and STAT3 phosphorylation. **(A)**, Relative mRNA expression of *STAT3* in CS AF-treated (n = 3) NCM460 intestinal epithelial cells compared to VD AF-treated group (n = 3); **(B)**, Immunoblotting analyses of the expression of STAT3 and p-STAT3 in colon of CS pups; **(C)**, IL-6 levels in AF samples of CS women (n = 7) compared to the VD women (n = 7); **(D)**, IL-10 and TNF-α levels in serum of CS-born cord blood from CS women (n = 7) compared to VD women (n = 7). Correlation analyses of AF vitamin D2 glucosiduronate and levels of IL-10 **(E)** and TNF-α **(F)** in the cord blood. Significance calculated with a two-tail student’s t test. Error bars represent SEM. *p < 0.05, **p < 0.01.

Genes differentially expressed by CS AF indicated that *STAT3* was significantly downregulated in CS AF treated NCM460 intestinal epithelial cells (**Figure 7A**). Immunoblotting confirmed the downregulation of STAT3 protein and STAT3 phosphorylation in colon of CS pups compared to VD pups (**Figure 7B**). Moreover, the levels of IL-6 (*p*=0.004) in AF samples were significantly lower in CS subjects compared to the VD subjects (**Figure 7C**). Additionally, lower levels of IL-10 (*p*=0.015) and TNF-α (*p*=0.032) were found in the cord blood of CS compared with VD subjects (**Figure 7D**). And vitamin D2 glucosiduronate with high abundance in VD AF showed a significant positive correlation to levels of IL-10 (*p*=0.007, **Figure 7E**) and TNF-α (*p*=0.012, **Figure 7F**) in the cord blood. Collectively, a lower level of vitamin D metabolite in CS AF was correlated with STAT3 signaling in both *in vitro* and *in vivo* animal and human samples.

## Discussion

Although epidemiological studies have linked the increasing use of CS to IBD, the causal effects of CS delivery on colonic inflammation and mechanisms underlying these effects in offspring are poorly understood. In this study, we found that mice delivered by CS exhibited a defective epithelial barrier and an increased susceptibility to DSS-induced colitis compared to VD pups. Multi-omics analyses using transcriptomics integrated with metabolomics demonstrated that metabolites with low abundances in human CS AF were correlated with DEGs in human intestinal epithelial cells treated with AF collected from CS patients at term. These genes were significantly enriched in pathways underlying IBD. Interestingly, lower levels of a vitamin D metabolite in CS AF was correlated with STAT3 signaling both *in vitro* and *in vivo* in animal and human samples, this may represent one of the underlying mechanisms of the association between CS and the risk of IBD in offspring. Overall, our findings may fill the knowledge gap of the role of CS-disrupted AF metabolites in intestinal inflammation and provide a novel insight into mechanisms underlying CS-originated disease.

Evidence suggests that the disruption of intestinal homeostasis linked by epithelial barrier function and mucosal immune cells is an important risk factor for IBD (24). An impaired gut barrier characterized by downregulation of the tight junction proteins, including ZO-1 and Occludin, and increases of serum zonulin have been observed in IBD (25, 26). In accordance, we found decreased gene expression of *ZO-1* and *Occludin* in the colons and increased levels of zonulin in serum of CS-delivered pups compared to VD pups before weaning, which indicates the disruption of the intestinal barrier integrity in CS-delivered pups during this stage. Moreover, the disturbed IEC differentiation has been linked to the develop of IBD (27), thus the generation of absorptive cells increases, the formation of secretory cells decreases, suggesting that CS may alter the IEC differentiation and disrupt the intestinal homeostasis. It is worth noting that no significant differences for the expression of tight junction markers (*ZO-1*, *Occludin*) and IEC markers (*Lyz1*, *Muc2*) in the colons were found at day 30. We speculate that breast feeding and an establishment of an adult-type microbiota signature progressively during growth may improve the disturbed intestinal barrier integrity. Studies have shown that gut microbiota composition and activity are involved in the regulation of the intestinal permeability (16, 28). Collectively, the disrupted intestinal homeostasis induced by CS may contribute to an increased susceptibility to DSS-induced colitis compared to VD pups.

An aberrant infiltration of immune cells to the mucosa can exacerbate inflammation in IBD patients (29–31). We herein detected an increased proportion of macrophages, DCs, and NK cells in the colons of CS-delivered pups at 10 days old. Recently, it has been demonstrated that intestine-resident macrophages and NK cells have a close association with epithelial cells and help maintain barrier integrity of the intestinal tract (32, 33). DCs are professional antigen presenting cells (APCs) with the ability to initiate adaptive immune responses against potential stimuli (34). Thus, the accumulation of these immune cells at 10 days old in our model might be a protective reaction of the host to distructive stimuli, such as components in AF. Strikingly, we observed dynamic changes in the immune cell populations in intestinal tissues during the growth of offspring. Specifically, we observed a decreased proportion of DCs, NK, and macrophages at 30 days old. Considering the protective role of DCs, NK, and macrophages in the intestine, the decreased proportions of these immune cells may progressively exacerbate colitis and cause weight loss and a shorter colon. Moreover, due to the absence of “trained and experienced” T and B cells, the neonatal gut exhibited a distinct immune system from adult (35). Macrophages and DCs are presented in low frequencies in neonatal gut, which may result in imperfect function and inactivity demonstrated by lower chemotaxis, reduced extravasation and a Th2 type response, and make neonates more vulnerable to infective agents (36). The frequencies of these monocytes increased and reaching adult levels by the establishment of gut microbiota and mature of immune system. Thus, the decreased proportions of these immune cells in CS colons compared to VD colons at postnatal day 30 might indicate a delay for the transition of immune cells from immature to mature. Collectively, our data suggest that CS induced imbalanced immune cells might be a high-risk factor for developing IBD.

Studies have showed that AF obtained from women delivered at term possesses anti-inflammatory property that makes it attractive for use in clinical applications (14, 37, 38). Given that these studies used sterile filtered AF and that the filtration process of AF eliminates cellular elements, hair, lanugo, and vernix from the fluid, we hypothesize that it is the soluble components of AF that are responsible for immunomodulatory and anti-inflammation processes. Data from our study revealed that the metabolites profiles of CS AF significantly differed from those of VD AF. These findings support our initial hypothesis that the composition of AF can be influenced by delivery mode. One of the limitations of our study is that we didn’t collect the stress and dietary data from mothers. Thus, we couldn’t eliminate the impact of maternal stress and diet during pregnancy on the composition of AF. Several products of glycerophospholipid metabolism (PC, PE, and PI et al.), which play an anti-inflammatory role in colitis (39) were significantly reduced in human CS AF. Comparison of transcriptomes between CS AF-treated and VD AF-treated IECs revealed a disturbance of genes involved in the lysosome, glycosaminoglycan degradation, complement and coagulation cascades, melanogenesis, and pentose phosphate pathway (PPP), all of which were associated with imminent development of CD (40). It is well documented that the fetus produces complement protein as early as the first trimester of development, and the complement cascade is fully developed at birth (41). However, decreased expression of *C3*, *C1S*, complement C1r (*C1R*), and plasminogen activator, urokinase receptor (*PLAUR*) were detected in CS AF-treated IECs, which indicates that the complement and coagulation cascades have not developed completely. IBD has a close association with transketolase (TKT), which links glycolysis and the PPP (42). The reduced expression of *TKT* in CS AF-treated IECs may disturb PPP metabolites and decreases glycolytic metabolites, result in a defective intestinal barrier (43). Thus, our data support the view that CS-induced discriminatory AF metabolites might contribute to the chronic intestinal inflammation in offspring.

It has been demonstrated that colonic vitamin D signaling activation protects the mucosal epithelial barrier and suppresses colonic inflammation (44). Vitamin D deficiency is associated with higher prevalence and worse outcomes from autoimmune diseases, including IBD (45). Notably, we found that vitamin D2 glucosiduronate (vitamin D2 metabolite) was dramatically decreased in CS AF than VD AF, with a fold-change of 19498. This massive difference in levels of vitamin D2 glucosiduronate suggests that it plays a role in intestinal inflammation mediated through CS AF. A recent study indicated that vitamin D promoted IL-10 *via* IL-6 signaling through induction of both STAT3 protein and STAT3 phosphorylation (46). In our study, a lower level of a vitamin D metabolite in CS AF is correlated with STAT3 signaling both *in vitro* and *in vivo* animal and human samples. Specifically, *STAT3* was significantly reduced in CS AF vs. VD AF-treated IECs, and a reduction of STAT3 protein and STAT3 phosphorylation was detected in colons of CS pups compared to VD pups at 20 days old. Proinflammatory cytokines are increased in maternal blood at term pregnancy and are associated with cervical ripening and the initiation of labor (47). Studies also reported higher levels of IL-6 and TNF-α in cord blood in vaginal delivery cases (48, 49). This is consistent with our observation that lower levels of IL-6 in CS AF and lower levels of IL-10 and TNF-α in cord blood collected from CS cases compared to VD cases. We speculate that vitamin D promoted IL-10 *via* IL-6 signaling through induction of both STAT3 protein and STAT3 phosphorylation. These data are in accordance with previously identified vitamin D/STAT signaling in intestinal inflammation. Moreover, we found that genes involved in vitamin digestion and absorption, such as solute carrier family 19 member 2 (*SLC19A2*), scavenger receptor class B member 1 (*SCARB1*), Wnt family member 6 (*WNT6*), and biotinidase (*BTD*) were significantly downregulated in CS AF-treated IECs. Taken together, our data suggest that the significantly lower level of the vitamin D metabolite, vitamin D2 glucosiduronate in CS AF might link the association between CS and intestinal inflammation in offspring. Vitamin D supplementation in children born by CS may be a potential intervention to reduce the risk of IBD. Functional studies of the discriminating metabolites in AF induced by CS, such as vitamin D2 glucosiduronate, are needed in further studies.

In conclusion, CS offspring exhibits an increased susceptibility to DSS-induced colitis in mice. Transcriptomics integrated with metabolomics analysis revealed that CS AF metabolites-induced DEGs in human intestine epithelial cells were significantly enriched in pathways underlying IBD. Deficiency of metabolites like vitamin D2 glucosiduronate in CS-AF may attribute to the risk of intestinal inflammation through blocking of STAT3 signaling in offspring. Our study provides novel insights into the underlying mechanisms and prevention strategies for CS-associated intestinal inflammation.

## Data availability statement

The raw data of RNA-seq for VD AF- and CS AF-treated NCM460 cells have been deposited in NCBI SRA database (PRJNA827852). Original data set and analysis script are available from authors upon request.

## Ethics statement

The studies involving human participants were reviewed and approved by Ethics Committees of Xinhua Hospital Affiliated to Shanghai Jiao Tong University School of Medicine. Written informed consent to participate in this study was provided by the participants’ legal guardian/next of kin. The animal study was reviewed and approved by Institutional Animal Care and Use Committee (IACUC) of Xinhua Hospital Affiliated to Shanghai Jiao Tong University School of Medicine.

## Author contributions

LF and JZ conceived and designed the experiments. YL performed sample processing and analyzed the data. YL drafted the manuscript. All authors reviewed the manuscript.

## Supplementary material

Supplementary Figure 1 Immunofluorescence staining of immune cells in colons of CS and VD pups. Representative immunofluorescence staining images of macrophage **(A)**, dendritic cells **(B)**, and NK cells **(C)** in colons of CS mice compared with VD mice.

## References

[1] Boerma T Ronsmans C Melesse DY Barros AJD Barros FC Juan L. Global epidemiology of use of and disparities in caesarean sections. Lancet (2018) 392:1341–8. doi: 10.1016/s0140-6736(18)31928-7 30322584

[2] Li H Luo S Trasande L Hellerstein S Kang C Li J. Geographic variations and temporal trends in cesarean delivery rates in china, 2008-2014. JAMA (2017) 317:69–76. doi: 10.1001/jama.2016.18663 28030701

[3] Liang J Mu Y Li X Tang W Wang Y Liu Z. Relaxation of the one child policy and trends in caesarean section rates and birth outcomes in china between 2012 and 2016: observational study of nearly seven million health facility births. BMJ (2018) 360:k817. doi: 10.1136/bmj.k817 29506980 PMC5836714

[4] Bager P Simonsen J Nielsen NM Frisch M. Cesarean section and offspring's risk of inflammatory bowel disease: a national cohort study. Inflammation Bowel Dis (2012) 18:857–62. doi: 10.1002/ibd.21805 21739532

[5] Ponsonby A Catto-Smith A Pezic A Dupuis S Halliday J Cameron D. Association between early-life factors and risk of child-onset crohn's disease among victorian children born 1983-1998: a birth cohort study. Inflammation Bowel Dis (2009) 15:858–66. doi: 10.1002/ibd.20842 19107784

[6] Li Y Tian Y Zhu W Gong J Gu L Zhang W. Cesarean delivery and risk of inflammatory bowel disease: a systematic review and meta-analysis. Scand J Gastroenterol (2014) 49:834–44. doi: 10.3109/00365521.2014.910834 24940636

[7] Sandall J Tribe RM Avery L Mola G Visser GHA Homer CSE. Short-term and long-term effects of caesarean section on the health of women and children. Lancet (2018) 392:1349–57. doi: 10.1016/s0140-6736(18)31930-5 30322585

[8] Cho C Norman M. Cesarean section and development of the immune system in the offspring. Am J Obstet Gynecol (2013) 208:249–54. doi: 10.1016/j.ajog.2012.08.009 22939691

[9] Martikainen M Keski-Nisula L Jakupović H Karvonen A Pekkanen J Hirvonen M. The lack of natural processes of delivery and neonatal intensive care treatment lead to impaired cytokine responses later in life. Am J Reprod Immunol (New York N.Y. 1989) (2017) 77(3). doi: 10.1111/aji.12621 28044379

[10] Nikiforou M Jacobs E Kemp M Hornef M Payne M Saito M. Intra-amniotic candida albicans infection induces mucosal injury and inflammation in the ovine fetal intestine. Sci Rep-UK (2016) 6:29806. doi: 10.1038/srep29806 PMC4944185 27411776

[11] Wolfs T Kallapur S Polglase G Pillow J Nitsos I Newnham J. IL-1α mediated chorioamnionitis induces depletion of FoxP3+ cells and ileal inflammation in the ovine fetal gut. PloS One (2011) 6:e18355. doi: 10.1371/journal.pone.0018355 21479249 PMC3066237

[12] Wolfs T Kramer B Thuijls G Kemp M Saito M Willems M. Chorioamnionitis-induced fetal gut injury is mediated by direct gut exposure of inflammatory mediators or by lung inflammation. Am J Physiol-Gastr L (2014) 306:G382–93. doi: 10.1152/ajpgi.00260.2013 PMC3949018 24458021

[13] Spencer J MacDonald T Finn T Isaacson P. The development of gut associated lymphoid tissue in the terminal ileum of fetal human intestine. Clin Exp Immunol (1986) 64:536–43. PMC1542427 3491695

[14] Underwood M Gilbert W Sherman M. Amniotic fluid: not just fetal urine anymore. J Perinatol (2005) 25:341–8. doi: 10.1038/sj.jp.7211290 15861199

[15] Lumbiganon P Laopaiboon M Gülmezoglu A Souza J Taneepanichskul S Ruyan P. World health organization global survey on maternal and perinatal health research group. method of delivery and pregnancy outcomes in asia: the WHO global survey on maternal and perinatal health 2007–08. Lancet (2010) 375:490–9. doi: 10.1016/S0140-6736(09)61870-5 20071021

[16] Liu Y Chen H Feng L Zhang J. Interactions between gut microbiota and metabolites modulate cytokine network imbalances in women with unexplained miscarriage. NPJ Biofilms Microbi (2021) 7:24. doi: 10.1038/s41522-021-00199-3 PMC7969606 33731680

[17] Liu Y Tang W Ao J Zhang J Feng L. Transcriptomics integrated with metabolomics reveals the effect of bisphenol f (BPF) exposure on intestinal inflammation. Sci Total Environ (2022) 816:151644. doi: 10.1016/j.scitotenv.2021.151644 34774955

[18] Caraux G Pinloche S. PermutMatrix: a graphical environment to arrange gene expression profiles in optimal linear order. Bioinformatics (2005) 21:1280–1. doi: 10.1093/bioinformatics/bti141 15546938

[19] Bolger A Lohse M Usadel B. Trimmomatic: a flexible trimmer for illumina sequence data. Bioinf (Oxford England) (2014) 30:2114–20. doi: 10.1093/bioinformatics/btu170 PMC4103590 24695404

[20] Kim D Langmead B Salzberg S. HISAT: a fast spliced aligner with low memory requirements. Nat Methods (2015) 12:357–60. doi: 10.1038/nmeth.3317 PMC4655817 25751142

[21] Anders S Pyl P Huber W. HTSeq–a python framework to work with high-throughput sequencing data. Bioinf (Oxford England) (2015) 31:166–9. doi: 10.1093/bioinformatics/btu638 PMC4287950 25260700

[22] Love M Huber W Anders S. Moderated estimation of fold change and dispersion for RNA-seq data with DESeq2. Genome Biol (2014) 15:550. doi: 10.1186/s13059-014-0550-8 25516281 PMC4302049

[23] Livak K Schmittgen T. Analysis of relative gene expression data using real-time quantitative PCR and the 2(-delta delta C(T)) method. Methods (San Diego Calif.) (2001) 25:402–8. doi: 10.1006/meth.2001.1262 11846609

[24] Sharma D Malik A Guy C Karki R Vogel P Kanneganti T. Pyrin inflammasome regulates tight junction integrity to restrict colitis and tumorigenesis. Gastroenterology (2018) 154:948–64.e8. doi: 10.1053/j.gastro.2017.11.276 29203393 PMC5847456

[25] Caviglia G Dughera F Ribaldone D Rosso C Abate M Pellicano R. Serum zonulin in patients with inflammatory bowel disease: a pilot study. Minerva Med (2019) 110:95–100. doi: 10.23736/s0026-4806.18.05787-7 30160088

[26] Kuo WT Zuo L Odenwald MA Madha S Singh G Gurniak CB. The tight junction protein ZO-1 is dispensable for barrier function but critical for effective mucosal repair. Gastroenterology (2021) 161:1924–39. doi: 10.1053/j.gastro.2021.08.047 PMC8605999 34478742

[27] Li C Zhou Y Rychahou P Weiss H Lee E Perry C. SIRT2 contributes to the regulation of intestinal cell proliferation and differentiation. Cell Mol Gastroentero Hepatol (2020) 10:43–57. doi: 10.1016/j.jcmgh.2020.01.004 PMC7210478 31954883

[28] Kosiewicz M Zirnheld A Alard P. Gut microbiota, immunity, and disease: a complex relationship. Front Microbiol (2011) 2:180. doi: 10.3389/fmicb.2011.00180 21922015 PMC3166766

[29] Geremia A Biancheri P Allan P Corazza G Di Sabatino A. Innate and adaptive immunity in inflammatory bowel disease. Autoimmun Rev (2014) 13:3–10. doi: 10.1016/j.autrev.2013.06.004 23774107

[30] Huang B Chen Z Geng L Wang J Liang H Cao Y. Mucosal profiling of pediatric-onset colitis and IBD reveals common pathogenics and therapeutic pathways. Cell (2019) 179:1160–76.e24. doi: 10.1016/j.cell.2019.10.027 31730855

[31] Larmonier C Shehab K Ghishan F Kiela P. T lymphocyte dynamics in inflammatory bowel diseases: Role of the microbiome. BioMed Res Int (2015) 2015:504638. doi: 10.1155/2015/504638 26583115 PMC4637034

[32] Poggi A Benelli R Venè R Costa D Ferrari N Tosetti F. Human gut-associated natural killer cells in health and disease. Front Immunol (2019) 10:961. doi: 10.3389/fimmu.2019.00961 31130953 PMC6509241

[33] Chikina AS Nadalin F Maurin M San-Roman M Thomas-Bonafos T Li XV. Macrophages maintain epithelium integrity by limiting fungal product absorption. Cell (2020) 183:411–28 e16. doi: 10.1016/j.cell.2020.08.048 32970988 PMC7646275

[34] Balan S Saxena M Bhardwaj N. Dendritic cell subsets and locations. Int Rev Cel Mol Bio (2019) 348:1–68. doi: 10.1016/bs.ircmb.2019.07.004 31810551

[35] Yu JC Khodadadi H Malik A Davidson B Salles E Bhatia J. Innate immunity of neonates and infants. Front Immunol (2018) 9:1759. doi: 10.3389/fimmu.2018.01759 30105028 PMC6077196

[36] Westrom B Arevalo Sureda E Pierzynowska K Pierzynowski SG Perez-Cano FJ. The immature gut barrier and its importance in establishing immunity in newborn mammals. Front Immunol (2020) 11:1153. doi: 10.3389/fimmu.2020.01153 32582216 PMC7296122

[37] Johnson HL Hazard JB Foisee PS O A. Amniotic fluid concentrate as an activator of peritoneal immunity. Surg Bynec Ostet (1936) 62:171–81. doi: 10.1186/s12974-016-0492-z

[38] Siggers J Ostergaard M Siggers R Skovgaard K Mølbak L Thymann T. Postnatal amniotic fluid intake reduces gut inflammatory responses and necrotizing enterocolitis in preterm neonates. Am J Physiol Gastrointest Liver Physiol (2013) 304:G864–75. doi: 10.1152/ajpgi.00278.2012 23518680

[39] Naito Y Takagi T Uchiyama K Katada K Yoshikawa T. Multiple targets of carbon monoxide gas in the intestinal inflammation. Arch Biochem Biophys (2016) 595:147–52. doi: 10.1016/j.abb.2015.06.020 27095232

[40] Torres J Petralia F Sato T Wang P Telesco S Choung R. Serum biomarkers identify patients who will develop inflammatory bowel diseases up to 5 years before diagnosis. Gastroenterology (2020) 159:96–104. doi: 10.1053/j.gastro.2020.03.007 32165208

[41] Zelek W Xie L Morgan B Harris C. Compendium of current complement therapeutics. Mol Immunol (2019) 114:341–52. doi: 10.1016/j.molimm.2019.07.030 31446305

[42] Ge T Yang J Zhou S Wang Y Li Y Tong X. The role of the pentose phosphate pathway in diabetes and cancer. Front Endocrinol (2020) 11:365. doi: 10.3389/fendo.2020.00365 PMC7296058 32582032

[43] Tian N Hu L Lu Y Tong L Feng M Liu Q. TKT maintains intestinal ATP production and inhibits apoptosis-induced colitis. Cell Death Dis (2021) 12:853. doi: 10.1038/s41419-021-04142-4 34535624 PMC8448773

[44] He L Liu T Shi Y Tian F Hu H Deb DK. Gut epithelial vitamin d receptor regulates microbiota-dependent mucosal inflammation by suppressing intestinal epithelial cell apoptosis. Endocrinology (2018) 159:967–79. doi: 10.1210/en.2017-00748 PMC5788002 29228157

[45] Holick M. Vitamin d deficiency. New Engl J Med (2007) 357:266–81. doi: 10.1056/NEJMra070553 17634462

[46] Chauss D Freiwald T McGregor R Yan B Wang L Nova-Lamperti E. Autocrine vitamin d signaling switches off pro-inflammatory programs of TH1 cells. Nat Immunol (2021) 23:62–74. doi: 10.1038/s41590-021-01080-3 34764490 PMC7612139

[47] Rhee K Goetzl L Unal R Cierny J Flood P. The relationship between plasma inflammatory cytokines and labor pain. Anesth Analg (2015) 121:748–51. doi: 10.1213/ane.0000000000000837 26097983

[48] Nandanan B Chua M Chiang W Goh A Kumar D Knippels L. Influence of mode of delivery on cytokine expression in cord blood. Hum Immunol (2019) 80:533–6. doi: 10.1016/j.humimm.2019.03.018 30926352

[49] Treviño-Garza C Villarreal-Martínez L Estrada-Zúñiga C Leal-Treviño M Rodríguez-Balderrama I Nieto-Sanjuanero A. Leptin, IL-6 and TNF-α levels in umbilical cord blood of healthy term newborns in relation to mode of delivery. J Obstet Gynaecol (2016) 36:719–21. doi: 10.3109/01443615.2016.1148128 27012976

