# Supplementary material for: Disrupted metabolic signatures in amniotic fluid associated with increased risk of intestinal inflammation in cesarean section offspring
Source: Front Immunol. 2023 Jan 24;14:1067602. doi: 10.3389/fimmu.2023.1067602 (PMC9903135; doi:10.3389/fimmu.2023.1067602)
Supplement: Supplementary file 1 [file Table_1.docx]

**Table S1.** **Disease activity index (DAI) scores**

| Weight loss (%) | Fecal shapes | Fecal blood | Score |
| --- | --- | --- | --- |
| 0 | Normal | Normal | 0 |
| 1~5 | Incompact | Occult | 1 |
| 6~10 |  |  | 2 |
| 11~15 | Loose | Bloody | 3 |
| >16 |  |  | 4 |
