# Supplementary material for: Disrupted metabolic signatures in amniotic fluid associated with increased risk of intestinal inflammation in cesarean section offspring
Source: Front Immunol. 2023 Jan 24;14:1067602. doi: 10.3389/fimmu.2023.1067602 (PMC9903135; doi:10.3389/fimmu.2023.1067602)
Supplement: Supplementary file 2 [file Table_2.docx]

**Table S2** Demographic and clinical characteristics of participants

| Variables | CS  (n = 7) | VD  (n = 7) | *P-value* |
| --- | --- | --- | --- |
| Maternal characteristics |  |  |  |
| Age (years) ^a^ | 31.6 ± 3.0 | 29.1 ± 3.6 | 0.20 |
| Pre-pregnancy BMI (kg/m^2^) | 24.2 ± 5.3 | 21.4 ± 4.3 | 0.19 |
| Gravida | 1.8 ± 1.0 | 1.6 ± 0.8 | 0.76 |
| Para | 1.5 ± 0.6 | 1.3 ± 0.5 | 0.56 |
| Gestational weeks | 38.9 ± 0.8 | 39.7 ± 1.2 | 0.22 |
| Bacterial vaginosis | 0 (0%) ^b^ | 0 (0%) | NE ^c^ |
| Colpomycosis | 0 (0%) | 0 (0%) | NE |
| Virus |  |  |  |
| HIV^+^ | 0 (0%) | 0 (0%) | NE |
| HPV^+^ | 0 (0%) | 0 (0%) | NE |
| Syphilis | 0 (0%) | 0 (0%) | NE |
| Gestational hypertension | 0 (0%) | 0 (0%) | NE |
| Gestational diabetes mellitus | 0 (0%) | 0 (0%) | NE |
| Preeclampsia | 0 (0%) | 0 (0%) | NE |
| Eclampsia | 0 (0%) | 0 (0%) | NE |
| Intrauterine infection | 0 (0%) | 0 (0%) | NE |
| Neonatal characteristics |  |  |  |
| Birth weight (g) | 3613 ± 230 | 3260 ± 361 | 0.08 |
| Gender |  |  |  |
| *Male* | 4 (57%) | 3 (43%) | 1.0 |
| *Female* | 3 (43%) | 4 (57%) | 1.0 |

a, Data are presented as mean ± SD;

b, Data are presented as n (%);

c. NE, not estimable (due to nullity of category in both groups).
