# Supplementary material for: Disrupted metabolic signatures in amniotic fluid associated with increased risk of intestinal inflammation in cesarean section offspring
Source: Front Immunol. 2023 Jan 24;14:1067602. doi: 10.3389/fimmu.2023.1067602 (PMC9903135; doi:10.3389/fimmu.2023.1067602)
Supplement: Supplementary file 3 [file Table_3.docx]

**Table S3** Primers used for RT-qPCR in this study

| Primer name | Tm (°C) | Sequence (5’-3’) |
| --- | --- | --- |
| Hu-C3-F | 60 | GGGGAGTCCCATGTACTCTATC |
| Hu-C3-R |  | GGAAGTCGTGGACAGTAACAG |
| Hu-C1R-F | 60 | GCTGACCTTCCACACAGACTT |
| Hu-C1R-R |  | CTCCCCTAATTTGCTCCGGG |
| Hu-C1S-F | 60 | TTTGGCATGGGTTTATGCTGA |
| Hu-C1S-R |  | GGGTGAAGTAGAGGTGAATCCC |
| Hu-ICAM5-F | 60 | CAGAGGGGTTTGCGTTGGTT |
| Hu-ICAM5-R |  | GAAAGTGCGAATGAGCCCAC |
| Hu-CD68-F | 60 | GGAAATGCCACGGTTCATCCA |
| Hu-CD68-R |  | TGGGGTTCAGTACAGAGATGC |
| Hu-PTGS1-F | 60 | CGCCAGTGAATCCCTGTTGTT |
| Hu-PTGS1-R |  | AAGGTGGCATTGACAAACTCC |
| Hu_HIF1A-F | 60 | GAACGTCGAAAAGAAAAGTCTCG |
| Hu_HIF1A-R |  | CCTTATCAAGATGCGAACTCACA |
| Hu_GPR1-F | 60 | CAATCTAGCCATTGCGGATTTCA |
| Hu_GPR1-R |  | CCGATGAGATAAGACAGGATGGA |
| Hu_DHRS2-F | 60 | CCTCTGGTAGGGAGCACTCT |
| Hu_DHRS2-R |  | CCAGCGCCACTACTGGATTA |
| Hu_RASGRP1-F | 60 | TGGAAACCTGTGTCGAAGTAAC |
| Hu_RASGRP1-R |  | ACTCCTCCATAGTGTCTGTCAAG |
| Hu_MAPK3-F | 60 | CTACACGCAGTTGCAGTACAT |
| Hu_MAPK3-R |  | CAGCAGGATCTGGATCTCCC |
| Hu_HYAL3-F | 60 | CTGTGCTGTGGAATGTACCCT |
| Hu_HYAL3-R |  | GTCATGTTCTGACCGTGAAAATG |
| Hu_FZD6-F | 60 | ATGGCCTACAACATGACGTTT |
| Hu_FZD6-R |  | GTTTACGACAAGGTGGAACCA |
| Hu_EP300-F | 60 | AGCCAAGCGGCCTAAACTC |
| Hu_EP300-R |  | TCACCACCATTGGTTAGTCCC |
| Hu_CD63-F | 60 | CAGTGGTCATCATCGCAGTG |
| Hu_CD63-R |  | ATCGAAGCAGTGTGGTTGTTT |
| Hu_SCARB1-F | 60 | CCTATCCCCTTCTATCTCTCCG |
| Hu_SCARB1-R |  | GGATGTTGGGCATGACGATGT |
| Hu_GAPDH-F | 60 | GGAGCGAGATCCCTCCAAAAT |
| Hu_GAPDH-R |  | GGCTGTTGTCATACTTCTCATGG |
| mZo1-F | 60 | GCCGCTAAGAGCACAGCAA |
| mZo1-R |  | TCCCCACTCTGAAAATGAGGA |
| mOcln-F | 60 | TTGAAAGTCCACCTCCTTACAGA |
| mOcln-R |  | CCGGATAAAAAGAGTACGCTGG |
| mLyz1-F | 60 | GAGACCGAAGCACCGACTATG |
| mLyz1-R |  | CGGTTTTGACATTGTGTTCGC |
| mMuc2-F | 60 | AGGGCTCGGAACTCCAGAAA |
| mMuc2-R |  | CCAGGGAATCGGTAGACATCG |
| mChgA-F | 60 | ATCCTCTCTATCCTGCGACAC |
| mChgA-R |  | GGGCTCTGGTTCTCAAACACT |
| mDpp4-F | 60 | ACCGTGGAAGGTTCTTCTGG |
| mDpp4-R |  | CACAAAGAGTAGGACTTGACCC |
| mVillin-F | 60 | TCAAAGGCTCTCTCAACATCAC |
| mVillin-R |  | AGCAGTCACCATCGAAGAAGC |
| mActin-F | 60 | CTACCTCATGAAGATCCTGACC |
| mActin-R |  | CACAGCTTCTCTTTGATGTCAC |
