# Supplementary material for: Disrupted metabolic signatures in amniotic fluid associated with increased risk of intestinal inflammation in cesarean section offspring
Source: Front Immunol. 2023 Jan 24;14:1067602. doi: 10.3389/fimmu.2023.1067602 (PMC9903135; doi:10.3389/fimmu.2023.1067602)
Supplement: Supplementary file 4 [file Table_4.docx]

**Table S4. Antibodies used in this study**

| REAGENT or RESOURCE | SOURCE | IDENTIFIER |
| --- | --- | --- |
| Antibodies for FC |  |  |
| Anti-mouse CD45 | BioLegend | Cat#: 103113 |
| Anti-mouse CD3 | BioLegend | Cat#: 100203 |
| Anti-mouse/human CD11b | BioLegend | Cat#: 101227 |
| Anti-mouse CD11c | BioLegend | Cat#: 117329 |
| Anti-mouse F4/80 | BioLegend | Cat#: 123129 |
| Anti-mouse NK-1.1 | BioLegend | Cat#: 108707 |
| Anti-mouse I-A/I-E (MHC Class II) | BioLegend | Cat#: 107613 |
| Anti-mouse CD16/32 | BioLegend | Cat#: 101319 |
| Antibodies for IF |  |  |
| Anti -F4/80 Rabbit pAb | Servicebio | Cat#: GB113373 |
| Anti-CD11c Rabbit pAb | ABclonal | Cat#: A1508 |
| Anti-CD11b Rabbit pAb | Servicebio | Cat#: GB11058 |
| Antibodies for WB |  |  |
| Anti-Occludin | Santa Cruz | Cat#: sc-133256 |
| Anti-ZO-1 | Santa Cruz | Cat#: sc-33725 |
| Anti-STAT3 | Santa Cruz | Cat#: sc-8019 |
| Anti-p-STAT3 | Santa Cruz | Cat#: sc-8059 |
| Anti-β-Actin | Santa Cruz | Cat#: sc-47778 |
| m-IgGκ BP-HRP | Santa Cruz | Cat#: sc-516102 |
