# Supplementary material for: Disrupted metabolic signatures in amniotic fluid associated with increased risk of intestinal inflammation in cesarean section offspring
Source: Front Immunol. 2023 Jan 24;14:1067602. doi: 10.3389/fimmu.2023.1067602 (PMC9903135; doi:10.3389/fimmu.2023.1067602)
Supplement: Supplementary file 5 [file Table_5.docx]

**Table S5** Characteristics of CS and VD pups

|  | Day10 | | | | Day20 | | | | Day30 | | | |
| --- | --- | --- | --- | --- | --- | --- | --- | --- | --- | --- | --- | --- |
|  | BW | | Sex | | BW | | Sex | | BW | | Sex | |
|  | VD | CS | VD | CS | VD | CS | VD | CS | VD | CS | VD | CS |
| N1 | 4.94 | 4.96 | ♂ | ♂ | 8.81 | 6.82 | ♀ | ♀ | 18.4 | 13.2 | ♀ | ♀ |
| N2 | 5.23 | 4.51 | ♂ | ♀ | 9.26 | 6.88 | ♀ | ♂ | 17.65 | 12.4 | ♂ | ♂ |
| N3 | 4.33 | 4.84 | ♂ | ♀ | 8.91 | 4.89 | ♂ | ♀ | 17.05 | 16.42 | ♂ | ♂ |
| N4 | 5.15 | 4.18 | ♂ | ♀ | 8.59 | 6.91 | ♀ | ♀ | 16.81 | 19.9 | ♂ | ♂ |
| N5 | 6.4 | 3.73 | ♀ | ♀ | 10.14 | 7.08 | ♂ | ♂ | 17.38 | 15.94 | ♂ | ♂ |
| N6 | 6.39 | 5.05 | ♀ | ♂ | 8.76 | 6.59 | ♂ | ♀ | 16.52 | 14.6 | ♀ | ♀ |

BW: body weight
