# Supplementary material for: Disrupted metabolic signatures in amniotic fluid associated with increased risk of intestinal inflammation in cesarean section offspring
Source: Front Immunol. 2023 Jan 24;14:1067602. doi: 10.3389/fimmu.2023.1067602 (PMC9903135; doi:10.3389/fimmu.2023.1067602)
Supplement: Supplementary file 6 [file Table_6.docx]

**Table S6** Linear regression analysis for body weight and sex in CS and VD pups

|  | β | 95% lower | 95% upper | P-value |
| --- | --- | --- | --- | --- |
| VD vs CS | 2.46 | 1.68 | 3.24 | 0.0002 |
| Female | -0.52 | -1.30 | 0.26 | 0.23 |
