# Supplementary figures and images for: Disrupted metabolic signatures in amniotic fluid associated with increased risk of intestinal inflammation in cesarean section offspring
Source: Front Immunol. 2023 Jan 24;14:1067602. doi: 10.3389/fimmu.2023.1067602 (PMC9903135; doi:10.3389/fimmu.2023.1067602)

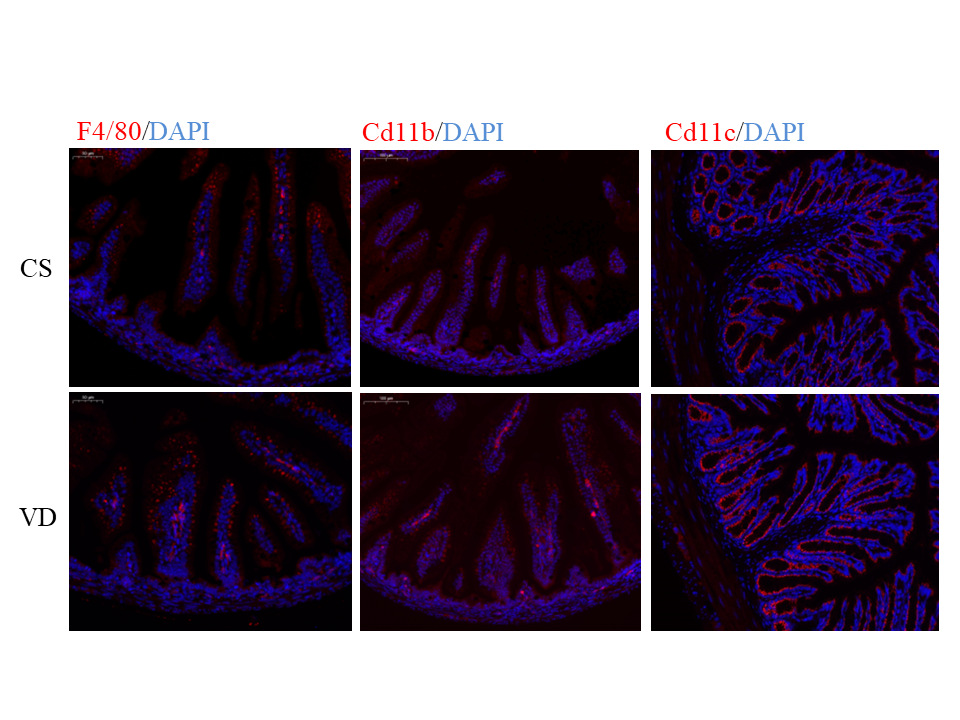

Supplement: Supplementary Figure 1 — Immunofluorescence staining of immune cells in colons of CS and VD pups. Representative immunofluorescence staining images of macrophage (A), dendritic cells (B), and NK cells (C) in colons of CS mice compared with VD mice. [file Image_1.tif]
